# Genetic analysis of MEFV mutation negative familial Mediterranean fever for non-MEFV mutations is rarely effective

**DOI:** 10.1186/1546-0096-13-S1-P26

**Published:** 2015-09-28

**Authors:** I Ben-Zvi, Y Shinar, R Cohen, C Grossman, O Kukuy, A Livneh

**Affiliations:** 1 Sheba Medical Center, Internal Medicine F, Ramat-Gan, Israel

## Background

Systemic autoinflammatory diseases (SAIDs) are a group of diseases characterized by episodes of inflammation, usually manifested with fever and a variety of symptoms, including skin-rash, arthritis and abdominal pain. A clinical overlap between different SAIDs, may cause diagnosis uncertainty. Familial Mediterranean fever (FMF), the prototype of the autoinflammatory syndrome, is manifested with recurrent attacks of fever and serositis. Although most FMF patients present with a typical picture, approximately 10% of them, present with atypical phenotype, and may harbor no mutations in their MEFV gene. In these patients further genetic analysis may be advocated.

## Objectives

In this study we aimed to study the frequency of gene mutations of 3 SAIDs in a population of atypical FMF patients.

## Patients and methods

By reviewing our records of FMF patients at Tel-Hashomer, we identified 10 patients with atypical FMF phenotype, who were non-responsive to colchicine, and carried no MEFV mutations. In these patients, we tested genetic mutations for TNF-receptor associated periodic syndrome (TRAPS), Hyper IgD Syndrome (HIDS), caused by Mevalonate kinase (MVK) deficiency and Cryopyrin associated periodic syndrome (CAPS).

## Results

Of the 10 patients who were recruited, 9 patients were found not to carry TRAPS, CAPS or HIDS mutations and 1 was heterozygous for the NLRP3 mutation K705Q, considered a non-pathogenic polymorphism. This patient had recurrent attacks of fever and skin-rash, without attacks of serositis. Since some patients with this polymorphism and SAID's symptoms were reported to respond to anti IL-1 treatment we offered our patients treatment with canakinumab, but she declined our advice.

## Conclusion

In FMF endemic area, screening of atypical FMF patients only rarely lead to a detection of another non-FMF SAID.

